# Drinking Among Young Adults: Screening, Brief Intervention, and Outcome

**Published:** 2004

**Authors:** Peter M. Monti, Tracy O’Leary Tevyaw, Brian Borsari

**Affiliations:** Peter M. Monti, Ph.D., is a senior research career scientist at the Providence Veterans Affairs Medical Center and a professor of medical sciences and director of the Center for Alcohol and Addiction Studies, Brown University, both positions in Providence, Rhode Island. Tracy O’Leary Tevyaw, Ph.D., is an assistant professor of psychiatry and human behavior, and Brian Borsari, Ph.D., is an assistant professor of community health, both at the Center for Alcohol and Addiction Studies, Brown University, Providence, Rhode Island

**Keywords:** young adult, undergraduate student, heavy drinking, binge drinking, alcohol dependence, problematic AOD (alcohol and other drug) use, AOD effects and consequences, harm reduction, identification and screening for AOD use, intervention (persuasion to treatment), brief intervention, motivational interviewing, school-based intervention, prison-based prevention, emergency room, medical-facility-based prevention, drinking and driving education, workplace-based prevention, Employee Assistance Program

## Abstract

Both college and noncollege populations face a high risk of becoming heavy drinkers and experiencing negative consequences of alcohol use. Because young people in these populations do not tend to identify themselves as having alcohol problems, they may be more readily identified through proactive screening in locations where they are likely to seek treatment related to alcohol problems, such as hospital emergency rooms, their college campuses, or workplaces. This article summarizes research on screening and brief interventions for alcohol use among young adults, particularly brief motivational interventions (BMIs).

Ample evidence demonstrates that excessive alcohol use among young people is a significant cause for public concern (see table). Young adults in the 18–25 age group consistently engage in high rates of risky behaviors such as unprotected sex and substance use (Arnett 2000). The National Survey on Drug Use and Health (Substance Abuse and Mental Health Services Administration [SAMHSA] 2003) revealed that young adults show the highest prevalence of problem drinking. Specifically, 41 percent of young adults reported drinking five or more drinks^1^ per occasion at least once in the past month (i.e., binge use), and 15 percent reported drinking five or more drinks per occasion on at least 5 different days in the past month (i.e., heavy use).^2^ Among college students, approximately 67 percent reported using alcohol at least once in the past month (Johnston et al. 2001), and 40 percent reported heavy episodic drinking (defined, for men, as drinking five or more drinks at least once in the past 2 weeks and, for women, four or more drinks at least once in the last 2 weeks) (Wechsler and Nelson 2001).

Heavy drinking also is prevalent among young adults in the military. In a survey of 5,136 military personnel ages 18 to 25, 2,763 (53.8 percent) reported a heavy-drinking episode in the past month, with 1,422 (28 percent) experiencing at least one heavy-drinking episode a week in the past month (Bray et al. 2003). Young adults often exhibit heavy drinking before entering the military; for example, 1,506 of 2,002 Navy recruits (89 percent under age 21) consumed alcohol in the past year, and one-third (519/1,506) of these recruits reported heavy episodic drinking as their typical consumption pattern (Ames et al. 2002).

**Table:** The Numbers Don’t Lie: The Need for Screening and Brief Intervention in the United States

**27,386**	Number of college students referred to school administration for violating school alcohol policy in 2001^1^
**99,109**	Number of first-time hospital emergency department visits that were alcohol related in 2001^2^
**6.2 million**	Number of full-time workers who are heavy alcohol users (five or more drinks on 5 or more days in the past month)^3^
**1,600**	Number of college students who die each year from injuries related to alcohol use^4^
**21.3%**	Percentage of driving under the influence (DUI) arrestees between the ages of 18 and 24^5^
**28%**	Percentage of parole violators under the influence of alcohol when committing a new offense^6^
**840,188**	Number of State and Federal inmates in need of substance abuse treatment^6^

1 Hoover 2003.

2 Hingson 2004.

3 SAMHSA 2002.

4 Hingson et al. 2005.

5 Bureau of Justice Statistics 1998.

6 Balenko 1998.

Among young adults, 25 percent of males and 14 percent of females meet (or at some time have met) the diagnostic criteria for alcohol dependence (SAMHSA 2003), as defined by the *Diagnostic and Statistical Manual of Mental Disorders, Fourth Edition* (DSM–IV) (American Psychiatric Association 1994), with 13 percent of men and 6 percent of women meeting diagnostic criteria for alcohol dependence in the past 12 months (Grant et al. 2004). However, many young people “mature out” of or moderate excessive drinking; as they enter their mid-twenties, their drinking no longer meets the criteria for abuse or dependence (Jackson et al. 2001). (For more discussion of this phenomenon, see the article by O’Malley in this issue.)

Despite the fact that young adults’ alcohol use is in some sense developmentally normative, combating heavy alcohol use in this population is important for several reasons. The primary causes of illness and death among young adults involve lifestyle and behavioral factors, including excessive alcohol use (Schulenberg et al. 2001). Even one episode of excessive drinking can have serious consequences that persist well beyond adolescence and young adulthood, such as alcohol-related car crashes, unintended pregnancies, and physical fights leading to arrest or jail. Young adults who engage in heavy episodic drinking are significantly more likely than those who do not drink heavily to get in trouble with police, damage property, sustain injuries, drive after drinking, and engage in unplanned and unprotected sexual activity (Wechsler et al. 2000). Alcohol use can trigger health problems even as early as adolescence and young adulthood. In one study, adolescents who drank heavily during their teen years were significantly more likely to be overweight and have high blood pressure by the time they were 24 years old (Oesterle et al. 2004). Finally, repeated instances of heavy episodic drinking among youth can have negative effects on the developing brain and brain function (Crews 2004; Caldwell et al. 2005; Monti et al. 2005) (see the article by Tapert and colleagues in this issue).

On college campuses, alcohol consumption consistently is linked to a variety of serious consequences. For example, in 1998 and 2001, more than 500,000 students were injured in drinking-related incidents, more than 600,000 were assaulted by a fellow student who had been drinking, and more than 1,600 students died each year from injuries related to alcohol use (Hingson et al. 2005).

In addition, neighbors of colleges who lived within 1 mile of campus were 135 percent more likely to report secondhand effects of college student drinking, including vandalism, assault, and other public disturbances (Wechsler et al. 2002*b*). Excessive alcohol use by college students is linked to risky sexual behavior (Wechsler et al. 2000). Among college women who reported being raped, 72 percent reported that the rape occurred while they were intoxicated (Mohler-Kuo et al. 2004). Alcohol consumption also is associated with academic impairment, including missing classes, doing poorly on tests, and getting behind in schoolwork (see Perkins 2002 for a review). Despite these risks, college students usually accept higher drinking levels than experts do when defining a drinking problem in a peer (Posavac 1993). They also tend to report feeling in control of their drinking even after acknowledging excessive alcohol use (Burrell 1992), and to dismiss the need to reduce heavy drinking although reporting alcohol tolerance and alcohol-related problems (Vik et al. 2000).

When they need medical attention for intoxication or injuries associated with alcohol use, many college students seek treatment off campus rather than campus-based medical care (Colby et al. 2000). As a result, college administrators may significantly underestimate the number of students who require medical attention for alcohol-related incidents. In addition, if intoxicated students drive themselves to off-campus medical facilities or rely on friends who have been drinking to drive them, they may risk further injury and harm.

A considerable number of students appear to require, but do not seek, medical treatment. A recent survey of more than 14,000 students revealed that 6.3 percent met DSM–IV criteria for alcohol dependence at some time over the past year, but only 6.2 percent of this 6.3 percent of students had sought treatment for their drinking (Knight et al. 2002). Some students may not seek medical help for illness or injuries related to drinking, or for alcohol dependence, because of concerns regarding confidentiality and administrative reprisals. For example, in one survey of 215 students, almost the entire sample reported having helped other students during or after an alcohol-related negative event, such as acute intoxication, passing out, and the like, and more frequently utilized off-campus medical help and local police rather than on-campus resources (Colby et al. 2000). These findings suggest that most college student drinking does not come to the attention of campus officials. Finally, in addition to a general lack of knowledge about the nature of drinking and related problems among young adults and among college students specifically, little is known about these problems among young adults who do not attend college.

## Screening

Because young adults do not tend to identify themselves as having alcohol problems, proactive screening should be conducted in locations where they are likely to present with alcohol-related injuries or illness. Among 18- to 24-year-olds, these environments may include hospital emergency departments (EDs), college counseling centers, college-sponsored judicial review programs for alcohol-related infractions of campus policies, and worksites.

### Hospital Emergency Departments

A study of 18- and 19-year-old patients who visited an ED for medical treatment found that patients who were treated for an alcohol-related event, such as a car crash, were significantly more likely to report more alcohol use and more alcohol-related problems than patients treated in the ED for events that did not involve alcohol (Barnett et al. 2003). This finding bolsters the notion that EDs are a useful venue for identifying young people who may have alcohol problems. Given the prevalence of alcohol use reported by young adults in the armed forces, it also would be beneficial for EDs on military installations or in the surrounding communities to screen military personnel who present with alcohol-related injuries.

Emergency departments use different methods to screen for alcohol use. One study examining young adults admitted to a trauma center through an ED found that 41 percent of the sample tested positive for alcohol, and more than 22 percent had blood alcohol concentrations in the legally intoxicated range (Rivara et al. 1992). The same study showed that among patients who had been drinking, a substantial proportion had injuries that stemmed from their alcohol use. However, research among young adults who present to emergency rooms has found that blood alcohol levels alone are not sufficiently reliable or sensitive to identify people with alcohol problems (e.g., Gijsbergs et al. 1991). A more comprehensive approach, using both screening instruments (Chung et al. 2002) and self-reports of alcohol consumption preceding the event that led to the ED visit (Monti et al. 1999), can be more useful in identifying alcohol problems.

### College Campus Venues

College counseling centers and health clinics can be other important venues for alcohol screening among young adults. O’Hare and Sherrer (1999) examined the validity and reliability of one screening measure, the Alcohol Use Disorders Identification Test (AUDIT), with college students who had violated campus alcohol policies. Their study found that the AUDIT was a psychometrically sound screening instrument for detecting harmful alcohol use in this population. In one large-scale screening of college students conducted as part of the 1999 National Alcohol Screening Day, 13,053 students were screened at 499 campuses (Greenfield et al. 2003). Of these students, 4,005 (31 percent) reported scores that were indicative of hazardous drinking. These findings support the idea that college campuses can provide an important context in which to identify young adults who are engaging in risky alcohol use. Nevertheless, some evidence indicates that few college health clinics routinely screen for alcohol and other drug use and abuse in students; Rickman and Mackey (1995) found that only 37 percent of 277 college student health clinics conducted such screening. Given the wide reach that student health clinics and counseling centers have on campuses, more efforts to screen students in these sites should yield valuable results.

### College Judicial Review Programs

Violations of campus alcohol policies lead to most of the judicial penalties colleges impose on students (Freeman 2001). From a prevention perspective, the need to screen students for alcohol use and misuse before they commit alcohol-related infractions is obvious. However, more and more researchers are recognizing the advantages of screening students who are mandated to attend alcohol education classes on campus as a penalty for violating college alcohol policy. This group is an appropriate target for alcohol screening because they already have begun to experience adverse consequences as a result of their drinking (that is, their referral to mandated education programs). In addition, the number of these “mandated students” is large and growing. In one survey of 199 schools, the number of students required to attend alcohol programs nearly doubled between 1993 and 2001 (i.e., from 1.8 percent to 3.5 percent of the schools’ student populations) (Wechsler et al. 2002*a*). Another recent study, which surveyed 4,711 2- and 4-year schools, indicated that the number of mandated students increased by 4.7 percent from 2000 to 2001 (Hoover 2003).

### On the Job

The workplace is another promising venue for screening young adults for alcohol-related problems. Researchers used the AUDIT as a workplace screening measure in a large-scale study of 4,193 law enforcement personnel conducted in Australia (Davey et al. 2000). Results indicated that participants between ages 18 and 25 reported the highest average alcohol consumption rates, and had higher rates of alcohol-related problems and higher AUDIT scores, than other age groups in the sample. Approximately one-third of the total sample was found to be at risk for hazardous alcohol consumption, further underscoring the desirability of screening young adults in the workplace.

## Brief Interventions

Traditional alcohol education programs, which provide information about the risks of alcohol use, have been implemented in a variety of ways (e.g., individual sessions, lectures, multisession groups). However, these approaches have not resulted in drinking reductions in either nonstudent or student populations (Hingson et al. 1997; Wells-Parker et al. 1995). Given the variety of drinking patterns evident in the young adult population and the minimal effect of traditional alcohol education programs, more targeted, systematic approaches are needed to help young adults recognize and reduce their hazardous drinking.

Young adults engaging in risky levels of alcohol use may respond more favorably to brief, more intensive interventions (Monti et al. 2001) than to traditional longer-term treatments, which originally were designed for adults with longer histories of alcohol use and alcohol-related problems (e.g., Monti et al. 2002). The precise definition of what constitutes a “brief intervention” has been the source of some debate (Moyer et al. 2002). Typically, brief interventions consist of one to four sessions with a trained interventionist (e.g., physician, psychologist, social worker), with each session ranging from 30 minutes to an hour. A recent meta-analysis (Moyer et al. 2002) of 34 studies found that people being treated for problems other than alcohol use (i.e., non-treatment-seeking participants) who received such brief interventions consistently showed greater reductions in alcohol use than did those assigned to no-treatment control groups. Among people seeking treatment for alcohol use, brief interventions and extended treatments (consisting of five or more sessions) were associated with similar reductions in alcohol use. Overall, the findings indicate that brief interventions can be an effective way to reduce drinking, especially among non-treatment-seeking people who do not have severe drinking problems that would require more intensive treatment.

A brief intervention that includes motivational interviewing is called a brief motivational intervention (BMI). BMI is a collaborative method that makes use of reflective listening and empathy as well as specific techniques (e.g., asking key questions, anticipating the future) to enable clients with alcohol-related problems to explore and resolve their ambivalence about reducing their alcohol use. This combination of reflective, empathic listening and specific techniques for change is known as motivational interviewing (MI).^3^ Interventions that use MI and incorporate other components are known as “adaptations of motivational interviewing” (AMIs) (Burke et al. 2003). Brief motivational interventions can be considered AMIs, as they often involve giving the client individualized feedback regarding his or her drinking and the risks associated with it.

In a meta-analysis of the clinical impact of BMIs on alcohol and marijuana use, Burke and colleagues (2003) found that 51 percent of clients who received BMIs reduced their substance use, compared with 37 percent of those who received assessment only or treatment as usual. For alcohol use specifically, BMIs showed large effects (average *d* = .82), with participants reducing their alcohol use by 56 percent (from 36 to 16 standard drinks per week) following a BMI. BMI effects also were maintained over time (as long as 4 years after treatment), even though BMIs took less time than the interventions with which they were compared.

BMIs are intended for people who do not have detectable signs or symptoms of a diagnosable disorder; as such, they can be considered “indicated preventive interventions” (see Mrazek and Haggerty 1994). People who are experiencing more severe problems (e.g., alcohol dependence) probably require more intensive treatment. Many BMIs have been effective in reducing alcohol use among nondependent adult drinkers, which suggests that they could be successfully implemented with young adults.

### Nonstudents

Although a significant percentage of young adults who are not college students engage in risky drinking and experience its consequences, BMIs have not yet been implemented extensively with this population. Several contexts appear promising for administering BMIs with young adults.

#### Emergency Departments

Hospital emergency departments are perhaps the places where young adults with drinking problems are most commonly identified. A study of 250 18- and 19-year-old patients in an urban ED found that nonstudents were at risk for alcohol use and problems, and that older adolescents tended to be more experienced drinkers (Barnett et al. 2003).

Fortunately, several studies have shown BMIs to be effective interventions with young adults who sought medical treatment in hospital emergency departments. Monti and colleagues (1999) randomly assigned 18- and 19-year-old ED patients to receive either BMI or standard care, which consisted of a handout on the hazards of drinking and of driving after drinking. During the intervention, a treatment provider assessed the participant at bedside with the help of a laptop computer that provided immediate personalized feedback on the participant’s drinking behavior. In addition to discussing this feedback, during the BMI the clinician and participant focused on the participant’s perceptions of norms regarding alcohol use; perceptions of the pros and cons of drinking, drinking and driving, and other alcohol-related risk behaviors; and expectations about alcohol use. The clinician also provided educational material about drinking. Followup data collected for 94 of the participants 6 months after the intervention demonstrated that the BMI group had significantly fewer alcohol-related injuries, traffic violations, and other alcohol-related problems than the standard-care group.

A more recent study (Monti and Barnett 2005) added two booster sessions to the BMI and compared this treatment with a feedback-only condition, which consisted of patients receiving the same baseline assessment and computer-generated personalized feedback sheet as did patients receiving a BMI. With participants receiving feedback only, counselors briefly stated that the sheet provided information from the assessment, but there was no further discussion. Two hundred and fifteen young adults were recruited into this trial. Results at 12 months show significant differences on several drinking variables such as number of drinking days and number of heavy-drinking days as well as on average blood alcohol concentrations, but no differences in alcohol-related consequences such as alcohol-related injuries and drinking and driving.

The results of these two studies show that emergency departments afford a unique opportunity to provide effective BMIs to young adults who engage in risky drinking.

#### Worksite Employee Assistance Programs (EAPs)

These programs, which have grown in number and use in the past 25 years, provide counseling and treatment at the worksite to workers who are experiencing problems, including problems related to alcohol and other substance abuse (Roman and Blum 2002). Given the variety of work settings and the types of problems encountered, the format of EAP interventions varies widely. Research indicates that when these programs focus on alcohol problems they can have positive results, enabling participants to reduce their alcohol use, especially when the EAP intervention includes strategies to prevent relapse (Roman and Blum 2002). About 80 percent of workers who enter EAPs do so on their own initiative. Most are women and are in their late thirties or early forties (French et al. 1997; Roman and Blum 2002). Little is known about the use of EAPs by younger adults, who tend not to seek any kind of treatment.

Because most young adults who are not attending college are employed full time, EAPs are a promising context for intervening with those who have developed problems with alcohol. EAPs may be particularly effective with this population if these programs include proactive alcohol screenings to identify these workers. The brief, nonconfrontational nature of BMIs may make them ideally suited for early indicated interventions with young workers who have not yet developed more serious alcohol problems.

#### DUI Programs

Almost 35 percent of people arrested for driving under the influence (DUI) are younger than 25 years old (McCarty and Argeriou 1988), and 10 percent are under age 21 (Socie et al. 1994). In addition, drivers younger than 21 years of age are at greater risk for repeat DUI arrests than older drivers (Socie et al. 1994). Because so many people arrested for drinking and driving are young, DUI treatment programs are a promising context for implementing BMIs. However, standard programs developed for DUI offenders, which range from group educational interventions to individual psychotherapy, require multiple sessions. A comprehensive meta-analysis of 215 studies of DUI programs revealed that standard interventions reduce repeat arrests by 8 to 9 percent (Wells-Parker et al. 1995), suggesting that DUI offenders can benefit from interventions aimed at changing behaviors. In recent years, DUI programs have increasingly targeted individual client attributes and needs (Williams et al. 2000). In one recent study of depressed adults, enhancing standard treatment with personalized feedback reduced repeat DUI arrests by 35 percent (Wells-Parker and Williams 2002).

#### Prison and Parole Programs

Young adults make up a large proportion of the U.S. prison population: A 1996 survey found that 34 percent of inmates were 24 years old or younger, and 75 percent of State inmates and 31 percent of Federal inmates require substance abuse treatment (Balenko 1998). As in EAPs and DUI programs, most young adults receiving substance abuse treatment in the prison system receive standard treatments, which might include self-help groups, individual and group counseling, therapeutic communities, and methadone maintenance (Balenko 1998).

Research indicates that providing substance abuse treatment during both incarceration and parole to those who need it is associated with reductions in criminal activity, substance use, and recidivism after release (e.g., Andrews et al. 1990). Younger parolees who use alcohol and other drugs also are at greater risk of recidivism (Balenko 1998; Zanis et al. 2003), and addressing the treatment needs of youthful offenders has been recognized as one of the challenges facing prison alcoholism programs (Valle and Humphrey 2002).

Methodological issues (e.g., treatment fidelity) have limited the conclusions that can be drawn from research implementing motivational interventions with incarcerated adults (see Ginsberg et al. 2002). However, several aspects of BMIs recommend their use with young adult offenders. Specifically, lack of motivation to change substance use has been recognized as a primary issue with this population (De Leon et al. 2000; Rosen et al. 2004). BMIs explicitly attempt to increase one’s motivation to reduce substance use. In addition, the warm, genuine, and empathetic style of motivational interviewing may be more acceptable than a confrontational approach for prisoners and parolees. Furthermore, providing prisoners and parolees with information in a nonjudgmental setting and permitting them to take an active role in the intervention may facilitate reductions in the use of alcohol and other drugs. Indeed, preliminary research with incarcerated young adults indicates that a brief motivational intervention enhanced treatment engagement (Stein et al. 2005).

### College Students

Much more research has been devoted to developing, implementing, and evaluating BMIs for college students than for nonstudents. In 1999, national concern regarding the widespread adverse effects of heavy alcohol use on college campuses led to the formation of the Task Force on College Drinking, convened by the National Institute on Alcohol Abuse and Alcoholism and consisting of educators, researchers, and students. (See the related sidebar by Robert Saltz, p. 249) In a review of the task force’s findings, Larimer and Cronce (2002) found BMIs to be more effective at reducing alcohol use among college students than other interventions (e.g., education programs). Therefore, BMIs delivered in one-on-one sessions continue to be developed and implemented for college students engaging in risky alcohol use.

#### Emergency Departments

Hospital emergency rooms are likely places for identifying alcohol problems among college students as well as nonstudents. In a recent study, students presenting to a hospital emergency room for both alcohol and non-alcohol-related problems were screened for alcohol use. Students who reported an AUDIT score of 6 or greater were invited to receive a brief open-ended counseling session (5–25 minutes). During the session, counselors used motivational interviewing techniques to help students examine their alcohol use and gave them a brochure addressing alcohol-related risks and providing a menu of strategies for reducing those risks. Three months later, these students demonstrated significant reductions in alcohol use, problems, and dependence symptoms, and more than 77 percent of participants viewed the BMI as somewhat or very helpful (Helmkamp et al. 2003). Although limited by the lack of a no-treatment control group, findings of this study indicate that BMIs may be an appropriate and effective treatment for college students taken to the ED for alcohol-related injuries.

#### College Campuses

Six published studies have evaluated BMIs provided to college students who have drinking problems. Baer and colleagues (1992) found that alcohol consumption dropped by up to 40 percent among college students who received 1 hour of feedback and advice using motivational interviewing, similar to those who participated in a 6-week skills training group. For both groups, these effects were maintained at the 2-year followup.

Marlatt and colleagues (1998) randomly assigned incoming college students who reported binge drinking or problems with alcohol use to a brief intervention (similar to the one used by Baer et al. 1992) or an assessment-only condition. Four-year followup showed that students in the brief intervention group experienced significant reductions in drinking rates and problems associated with alcohol, compared with their own baseline levels and with students in the assessment-only condition (Baer et al. 2001). This study produced a manual, *Brief Alcohol Screening and Intervention for College Students* (BASICS), describing the brief intervention these researchers used (Dimeff et al. 1999). Borsari and Carey (2000) implemented the BASICS approach at a Northeastern university; compared with an assessment-only control group, BMI participants showed significant reductions in alcohol use at 6-week followup.

Three more recent evaluations compared BMIs with other active interventions. Murphy and colleagues (2001) compared a BMI (using the BASICS protocol) with an assessment-only condition and an individualized educational intervention that consisted of watching a video detailing alcohol-related risks. Although participants in the three conditions showed no overall significant differences in alcohol use at the 3- and 9-month followups, BMI participants who drank 25 or more drinks per week (i.e., heavy drinkers) reduced their weekly alcohol consumption and binge drinking by greater amounts than did heavy drinkers in the other two groups.

In a study of college fraternity members, Larimer and colleagues (2001) compared (a) a one-on-one BMI paired with a 1-hour group feedback session provided to the whole fraternity with (b) a 1-hour didactic presentation on alcohol use, with no personalized feedback. At the 1-year followup, students who had received the BMI reported greater reductions in average use and self-reported typical peak blood alcohol content. No reductions in alcohol-related consequences were observed.

Finally, in a study of heavy-drinking college students, Murphy and colleagues (2004) provided personalized feedback with or without a motivational interviewing session. A 6-month followup revealed significant, small-to-moderate reductions in alcohol use, but no differences between the groups and no change in alcohol-related problems for either group.

Overall, this research indicates that personalized feedback and motivational interviewing appear to influence changes in drinking behaviors and, to a much lesser extent, alcohol-related problems.

#### Mandated Students

Mandated students are students who have violated campus alcohol policies. Given that these students are often the heavier drinkers on campus, several projects using BMIs with mandated students have been implemented in the past 5 years. For example, Borsari and Carey (in press) randomly assigned mandated students to receive either a 60- to 90-minute motivational interview (BMI) or a 60- to 90-minute alcohol education session in which the student was provided information about alcohol and its effects. Following their referral incident, all eligible participants had continued to binge drink (defined as having had two or more binge-drinking episodes in the past month). At 3- and 6-month followups, both treatment groups demonstrated significant drinking reductions, with BMI students reporting significantly fewer alcohol-related problems than the alcohol education students at the 6-month followup.

Recently, adaptations of BMIs for mandated students have been developed (see Barnett et al. 2004). For example, BMIs incorporating booster sessions have been compared with a 45-minute interactive computer program (Alcohol 101 Plus) that provides the student with information about the effects of alcohol and the risks associated with excessive alcohol use (Century Council 2003). In addition, students in both conditions were randomly assigned to receive booster sessions 1 month after the intervention. The booster sessions were a shorter version of the original intervention, lasting 25 to 30 minutes. Reductions in alcohol use were evident in both groups at a 3-month followup, suggesting that both approaches may be valuable in reducing drinking in mandated students. In addition, students who received a BMI with a booster session were most likely to seek further assistance, suggesting that face-to-face contact in BMI may facilitate problem recognition in mandated students.

Another BMI adaptation involves the active participation of a peer of the mandated student in the intervention. This peer, selected by the mandated student, also receives personalized feedback about his or her own alcohol use and supports the student’s goals for reducing hazardous alcohol use (O’Leary et al. 2002). Although this study did not include a control condition, results indicated that students receiving peer-enhanced BMIs reported reductions in alcohol use similar to those reported by students receiving standard BMIs.

Taken together, these findings demonstrate the flexibility of BMIs and indicate that they are an effective option for campus alcohol programs intended to reduce heavy episodic drinking in mandated students.

## Conclusions

Excessive alcohol use among young adults is a major public health concern. Although drinking among college students has received the most research attention, it is a problem among noncollege students as well. Young adults in both groups rarely identify themselves as problem drinkers, which suggests that proactive screening approaches may be warranted. Several screening methods recently have proven effective and deserve further research attention.

Most interventions studied with young problem drinkers have incorporated BMIs. Promising contexts in which to implement BMIs with nonstudents include hospital EDs, EAPs, DUI programs, and prison- and parole-based programs. Among college populations, where more research on BMIs has been conducted, outcomes are impressive. Convincing results have been obtained for BMIs with students whose alcohol problems were identified in emergency departments and with students from the general college population who were identified as having drinking problems. In general, findings suggest that personalized feedback and motivational interviewing influence change in drinking variables and, to a lesser degree, in alcohol-related problems. Results with students mandated to alcohol treatment demonstrate the effectiveness of BMIs in reducing heavy episodes of alcohol use and alcohol-related problems.

BMIs have been implemented in a variety of contexts, with varying ranges of alcohol use and problems, and with both treatment-seeking and non-treatment-seeking populations. The flexibility and effectiveness of BMIs make them a promising component of stepped care, in which people first are assigned to the least restrictive, intrusive, and costly treatment that has a good chance of success and, if they do not respond to this initial level of treatment, are provided more intensive care (Borsari and Tevyaw 2005; Sobell and Sobell 2000). Thus, BMIs could address different degrees of alcohol use and problems by serving as a stand-alone intervention for people with less severe alcohol problems or as an initial screening and intervention tool for people who will require more intensive treatment.

Despite the promise of BMIs, further research is needed to determine precisely how these interventions facilitate behavior change. For example, which interviewer and client in-session behaviors are related to change (e.g., Amrhein et al. 2003)? Furthermore, does the inclusion of significant others or peers (e.g., O’Leary et al. 2002; Tevyaw et al. 2005) enhance the BMI session for the participant? If so, what is the responsible mechanism? Addressing these and other research questions likely will improve the efficacy of BMIs in addressing alcohol use and problems in young adults.

## Footnotes

1 The National Institute on Alcohol Abuse and Alcoholism (NIAAA) defines a standard drink as about 14 g of alcohol, which corresponds to approximately 1.5 oz of 80-proof distilled spirits, one glass of wine, or one 12-oz beer or wine cooler.

2 These definitions of heavy drinking and binge drinking differ from those provided by NIAAA, which defines binge drinking as consumption of 4 or more drinks in about 2 hours for women, and 5 or more drinks for men; heavy drinking is defined as more than 7 drinks per week for women and more than 14 drinks per week for men.

3 Motivational interviewing is a client-centered counseling style to help clients change their behavior by enabling them to deal with their ambivalence about the change (Miller and Rollnick 2002). MI can be either directive (e.g., when selectively eliciting and discussing possible change) or nondirective (e.g., when exploring ambivalence and maintaining a neutral stance regarding the person’s options).
